# Exploring Nanoscale Lubrication Mechanisms of Multilayer MoS_2_ During Sliding: The Effect of Humidity

**DOI:** 10.3389/fchem.2021.684441

**Published:** 2021-06-24

**Authors:** Victor E. P. Claerbout, Paolo Nicolini, Tomas Polcar

**Affiliations:** Department of Control Engineering, Faculty of Electrical Engineering, Czech Technical University in Prague, Prague, Czechia

**Keywords:** molybdenum disulfide, friction, hydrogen bond network, molecular dynamics simulations, tribology, water, humidity

## Abstract

Solid lubricants have received substantial attention due to their excellent frictional properties. Among others, molybdenum disulfide (MoS_2_) is one of the most studied lubricants. Humidity results in a deterioration of the frictional properties of MoS_2_. The actual mechanism at the nanoscale is still under debate, although there are indications that chemical reactions are not likely to occur in defect-free structures. In this study, we performed nonequilibrium molecular dynamics simulations to study the frictional properties of multilayer MoS_2_ during sliding in the presence of water. Moreover, we also investigated the effect of sliding speed and normal load. We confirmed earlier results that a thin layer of water organizes as a solidified, ice-like network of hydrogen bonds as a result of being confined in a two-dimensional fashion between MoS_2_. Moreover, we found that there exists an energy-driven, rotational dependence of the water network atop/beneath MoS_2_. This orientational anisotropy is directly related to the dissipative character of MoS_2_ during sliding. Finally, three distinct frictional regimes were identified, two for a thin layer of water and one for bulk water. In the case of a thin layer and low coverage, water represents a solid-like contaminant, causing high energy dissipation. For a thin layer and high coverage, water starts to act as a solid-like lubricant, reducing dissipation during sliding. Finally, a regime where water acts as a liquid lubricant, characterized by a clear velocity dependence was found.

## 1 Introduction

The presence of friction and wear has been linked to a loss of almost a quarter of the world’s total energy consumption (Holmberg and Erdemir, 2017). The current demand of our industry has surpassed the application of “classical” liquid lubricants in peculiar conditions, such as ultrahigh vacuum, extreme contact pressures, super low/high temperatures, and extremely small dimensions. A new generation of lubricants, the so-called solid lubricants, offers new possibilities. Moreover, solid lubricants have been linked to superlubricity, a state in which the frictional forces vanish (Baykara et al., 2018; Martin and Erdemir, 2018). Among others, transition metal dichalcogenides (TMDs), and in particular molybdenum disulfide (MoS_2_), are prototypical examples. TMDs are layered materials, where the monolayers have the so-called two-dimensional (2D) crystal structure with a transition metal covalently bound between chalcogens. The layers are held together by weak electrostatic and van der Waals interactions. As a result of this unique binding character, easy shearing between the layers is accommodated, creating an enormous potential for achieving low coefficients of friction (COF) (Martin et al., 1993; Scharf and Prasad, 2013). This is confirmed by numerous studies on their remarkable tribological characteristics (Martin et al., 1993; Watanabe et al., 2004; Cho et al., 2006; Evaristo et al., 2008; Pimentel et al., 2011; Mutafov et al., 2015). This, in combination with their low toxicity (Teo et al., 2014; Chng and Pumera, 2015), versatile chemistry (Chhowalla et al., 2013), and peculiar electronic properties, has resulted both in macroscale applications, such as the automotive, aerospace, and space industries (Cho et al., 2006; Nian et al., 2017; Vazirisereshk et al., 2019) and nanoscale applications, such as photovoltaic and optoelectronic devices, transistors, and solar cells (Li and Zhu, 2015; Choi et al., 2017).

In a significant number of (potential) applications, ambient conditions, such as humidity, are unavoidable, resulting in contaminated surfaces. Humidity however has shown to induce a significant alteration in the tribological properties of TMDs and other 2D materials, such as graphite/graphene (Evaristo et al., 2008; Scharf and Prasad, 2013; Levita et al., 2016; Levita and Righi, 2017; Hasz et al., 2018). In the case of MoS_2_, humidity not only leads to an increase in wear but also to a significant degradation in frictional properties (Panitz et al., 1988; Chhowalla and Amaratunga, 2000; Zhao and Perry, 2010; Khare and Burris, 2013; Levita and Righi, 2017; Serpini et al., 2017; Arif et al., 2019; Serpini et al., 2019). For example, it has been found that clusters of contaminating adsorbates can induce a static friction, seemingly “locking” surfaces in relative motion that would otherwise slide smoothly (Ouyang et al., 2018). As a result, MoS_2_ solid lubrication currently has a relatively small effective working range, limited to outer space or vacuum (Hasz et al., 2018). To extend its applicability, one should fully understand how humidity affects the lubricating mechanisms at the nanoscale during sliding.

Although the scientific community agrees that the beneficial frictional properties of MoS_2_ deteriorate in case of increasing humidity (Zhao and Perry, 2010; Dudder et al., 2011; Khare and Burris, 2014; Serpini et al., 2017), the actual mechanism at the nanoscale is still under debate (Hasz et al., 2018). Some ascribe it to oxidation, whereby MoS_2_ oxidizes to MoO_2_ (Panitz et al., 1988; Tagawa et al., 2007; Curry et al., 2017). Others showed that humidity does not necessarily lead to oxidation but that water physically interacts with the surface, thereby increasing interlayer friction (Khare and Burris, 2013; Levita and Righi, 2017; Serpini et al., 2017; Arif et al., 2019). Some use an argument based on adhesion, when explaining the changing frictional properties as a result of humidity (Hasz et al., 2018). In an experimental study, Lee et al., (2020) showed that when a layer of water is intercalated between mica and exfoliated MoS_2_, the frictional properties deteriorated. Interestingly, similar results were found for graphene and mica (Lee et al., 2017), contradicting previous reports that humidity leaves the frictional behavior unchanged (Berman et al., 2014). These results hint at a universal frictional mechanism for intercalated water layers that is independent of the type of solid lubricant itself (Lee et al., 2020).

The frictional response of a material is highly complex and characterized by nonlinear, out-of-equilibrium dissipative processes (Vanossi et al., 2013). Simulation techniques offer possibilities to gain insights by disentangling the phenomena and using simplified models. Among others, Levita et al. made several computational contributions (Levita et al., 2016; Levita and Righi, 2017). In one study (Levita et al., 2016), the type of interaction between MoS_2_ and water was determined using first-principle calculations (FPCs). The literature was discordant on whether this should be depicted as hydrophobic or hydrophilic (Late et al., 2012; Gaur et al., 2014). In addition, the tendency of MoS_2_ toward “adsorption of” vs. “being oxidized by” water was investigated. Due to the stable basal plane of MoS_2_ (lacking any dangling bond and characterized by low chemical reactivity), it was found that also in case of defects, such as S-vacancies, only physisorption occurs. This observation was confirmed by others (Arif et al., 2019). Upon introducing water within the bilayer interface, the interlayer distance was found to increase, weakening the interlayer binding energy and destabilizing the structure. It was confirmed that MoS_2_ does not favor oxidation (e.g., to MoO_2_) and acts rather hydrophobically (Levita et al., 2016). This conclusion has been shared by others (Luan and Zhou, 2016) using molecular dynamics (MD) simulations.

To study the frictional properties, sliding should be considered; after all, kinetic friction is a dynamical response. In another study, based on *ab initio* MD, the chemical and physical interactions taking place during sliding of a single asperity of MoS_2_, in the presence of water, were studied (Levita and Righi, 2017). At high temperature and load, it was found that increasing water coverage present within the interface reduces the distance traversed after an initial kick. Again, and as expected from previous studies (Khare and Burris, 2013; Levita et al., 2016), no oxidation occurred. Furthermore, it was found that the water molecules formed a water network of H bonds. Finally, S-vacancies did not induce significant deviations in the results. These observations were confirmed mainly by an experimental study (Arif et al., 2019), indicating that the main mechanism affecting the frictional properties of MoS_2_, in the presence of water, is physisorption.

The earlier mentioned results are relevant, but some remarks are in place. Levita and Righi (2017) used a high load (11 GPa) and temperature (320 K) to provide enough energy to the system to overcome potential energy barriers, which might affect the outcomes. Additionally, the simulation time was limited to the picosecond range, affecting the statistical precision of the results. Moreover, a structural analysis of the water network was not properly considered. When liquids, and water in particular, are confined to a small space, their properties might alter significantly (Bampoulis et al., 2016); this is in fact one of the problems of classical lubricants. In the case of water confined between two hydrophobic layers, ice-like water films have been found (Bampoulis et al., 2016), raising the question whether water acts as a (solid) lubricant or as a friction inducer (Lee et al., 2020). In the case of MoS_2_, Kwac et al. (2017) found that using MD, a characteristic tile-like pattern of squares and diamond forms, resulting from the MoS_2_ surface charge. However, the sliding of the MoS_2_ layers was not considered in this study.

The main aim of this study is to present a qualitative and quantitative analysis of the nanoscale frictional properties when sliding multilayer MoS_2_ in the presence of water. More generically, our goal is to reveal broader features of the frictional behavior of solid lubricants in the presence of contaminants. Based on previous results (Khare and Burris, 2013; Levita and Righi, 2017), tribochemical reactions were not expected to occur. Therefore, classical nonequilibrium MD simulations were performed. This was done by considering the intercalation of various number of water molecules, varying from dry sliding to bulk water, located at the center interface. Furthermore, the effects of normal load and of sliding speed were investigated. Apart from gaining insights into the conformational landscape of water, this setup allows for insights into the mechanisms of the additional channel of dissipation, present when considering intercalated water, in the case of bulk MoS_2_ and that of solid lubricants in general.

## 2 Computational Methods

**FIGURE 1:**
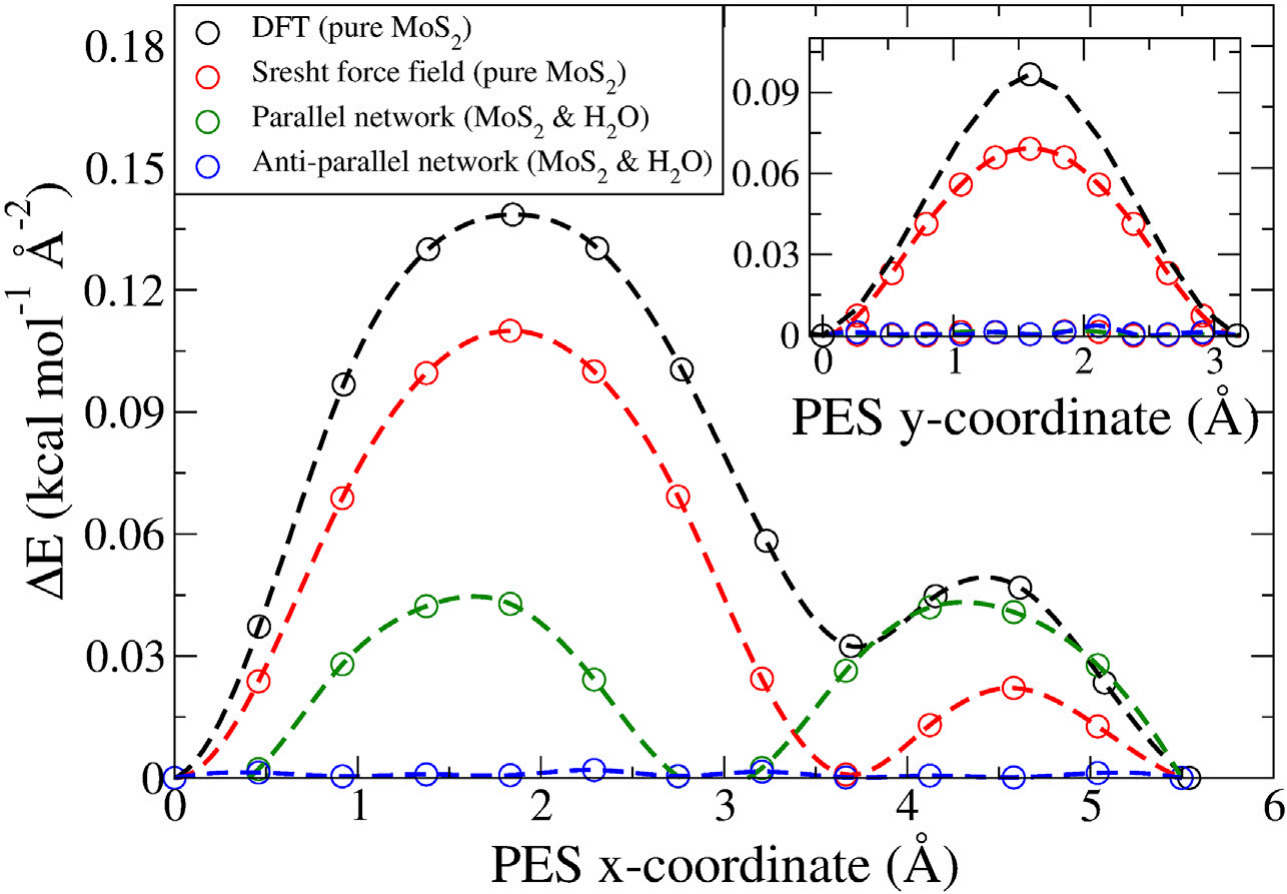
Static potential energy surfaces calculated per area for the long (inset: short) diagonal of the hexagonal unit cell. Four different cases are considered: i) in black, pure MoS_2_ obtained *via* DFT (Levita et al., 2014), ii) in red, pure MoS_2_ obtained with the force field employed in this study (Sresht et al., 2017), iii) in green, MoS_2_ and an ideal, parallel water network *via* MD, and iv) in blue, MoS_2_ and an ideal, antiparallel water network *via* MD. The dashed lines are obtained *via* akima spline interpolation and serve merely as a guide to the eyes.

The simulations have been carried out by means of the LAMMPS package (Plimpton, 1995), employing a force field developed by Sresht et al. (2017) for MoS_2_. Its foundation is based upon harmonic bonds and angles, with partial charges set to Mo=+0.50e and S=−0.25e , in combination with the Lennard–Jones (LJ) potential (Sresht et al., 2017). Water was modeled through a nonpolarizable q-SPC/Fw model (Paesani et al., 2006), characterized by a three-site water molecule, where the charges were set to O=−0.84e and H=+0.42e . Using Lorentz–Berthelot mixing rules, $\sigma_{ij}=(\sigma_{ii}+\sigma_{jj})/2$ and $\epsilon_{ij}=\sqrt{\epsilon_{ii}\epsilon_{jj}}$ , cross-term parameters were obtained. A cutoff of 12 Å was used for the LJ potential. To account for electrostatic interactions, the particle–particle particle–mesh (PPPM) method was used, with a threshold of $1.0\times 10^{-6}$ for the relative error in the forces. In Figure 1 and in the Supplementary Material, a benchmark of this force field is presented against FPC results (Levita et al., 2014) and experimental data (Schönfeld et al., 1983), by means of the sliding potential energy surface (PES) and structural parameters, respectively. The PES was obtained by performing static calculations on a partially rigid MoS_2_ bilayer. Here, only the top layer was allowed to breathe in the *z*-axis and was translated along the *x*-axis and the *y*-axis in twelve steps, for a total distance equal to the size of the period in that direction. (For more details, we refer to Nicolini and Polcar (2016).). The employed force field shows good agreement with both *ab initio* and experimental data.

First, the six-layered 2H_*c*_-MoS_2_ system was built using the initial coordinates from Schönfeld et al. (1983). The resulting structure consists of 4,608 atoms and a surface of approximately 44 Å by 51 Å. An energy minimization was performed using a sequence of conjugate gradient (CG) algorithm, with a relative energy tolerance of $1\times 10^{-15}$ , and damped dynamics (DD) algorithm, with a relative energy tolerance of $1\times 10^{-10}$ ; the simulation box was also allowed to relax. Second, an isolated water molecule was optimized according to the force field using CG. The resulting structure was replicated in three dimensions to create a large cube, with a density equivalent to that of liquid water at room temperature, and optimized using both CG and DD, while relaxing the box. Third, the cube of water was put atop three layers of MoS_2_ and subsequently optimized using both CG and DD. Thereafter, the water cube was heated up to 300 K using the Nose–Hoover thermostat (Martyna et al., 1994), with a temperature damping parameter of 100 time steps. In the fourth step, the actual structures were created. Random clusters of water molecules of the melted cube, located within a 5 Å cutoff above the nearest sulfur layer, were selected. The reason that we chose clusters, instead of randomly spread water molecules, had to do with the fact that in preliminary simulations, we found that the sliding of MoS_2_ drives the formation of clusters and therefore are the best representation. Finally, the missing three top layers of MoS_2_ were pasted above. By considering the “tiled” structure of the water network mentioned in Kwac et al. (2017), full coverage requires 304 H_2_O molecules. In total, 11 different systems were created consisting of 0 (dry), 1, 2, 5, 10 (3% coverage), 26 (8.5%), 52 (17%), 103 (33%), 206 (67%), 304 (100% full coverage), or 1,236 (bulk) water molecules. Finally, a vacuum of 20 Å was added above each structure in order to avoid interaction with the repeated images while applying periodic boundary conditions.

After obtaining the structures, all were once more optimized using CG and DD, while keeping the box dimensions fixed. This time, the bottommost sulfur layer was held fixed in the *x*-, *y*-, and *z*-direction. Moreover, the topmost sulfur layer was made rigid in the xy-plane and the forces in the *z*-direction were averaged over all sulfur atoms in this layer. This restriction on the top sulfur layer was added to mimic adhesion to the substrate and to be able to control the sliding. After the minimization, a 2-step equilibration was performed, in which seven configurations of every system were created, each with a different normal load applied to the topmost sulfur layer. The normal load was ranging from 0 to 3.0 GPa, with increments of 0.5 GPa. In the first step (100 ps), the load was slowly increased from 0 GPa to the target value, whereas in the second step (100 ps), it was held constant. During the equilibration, the system was thermalized by initializing the temperatures for every atom using the Boltzmann distribution. Moreover, the bottom and top layers of MoS_2_ were coupled to a Nose–Hoover thermostat (Martyna et al., 1994). On the one hand, this allows for maintaining the temperature during equilibration, while on the other hand, this avoids overheating from Joule heat production during the actual sliding runs (Vanossi et al., 2013).

**FIGURE 2:**
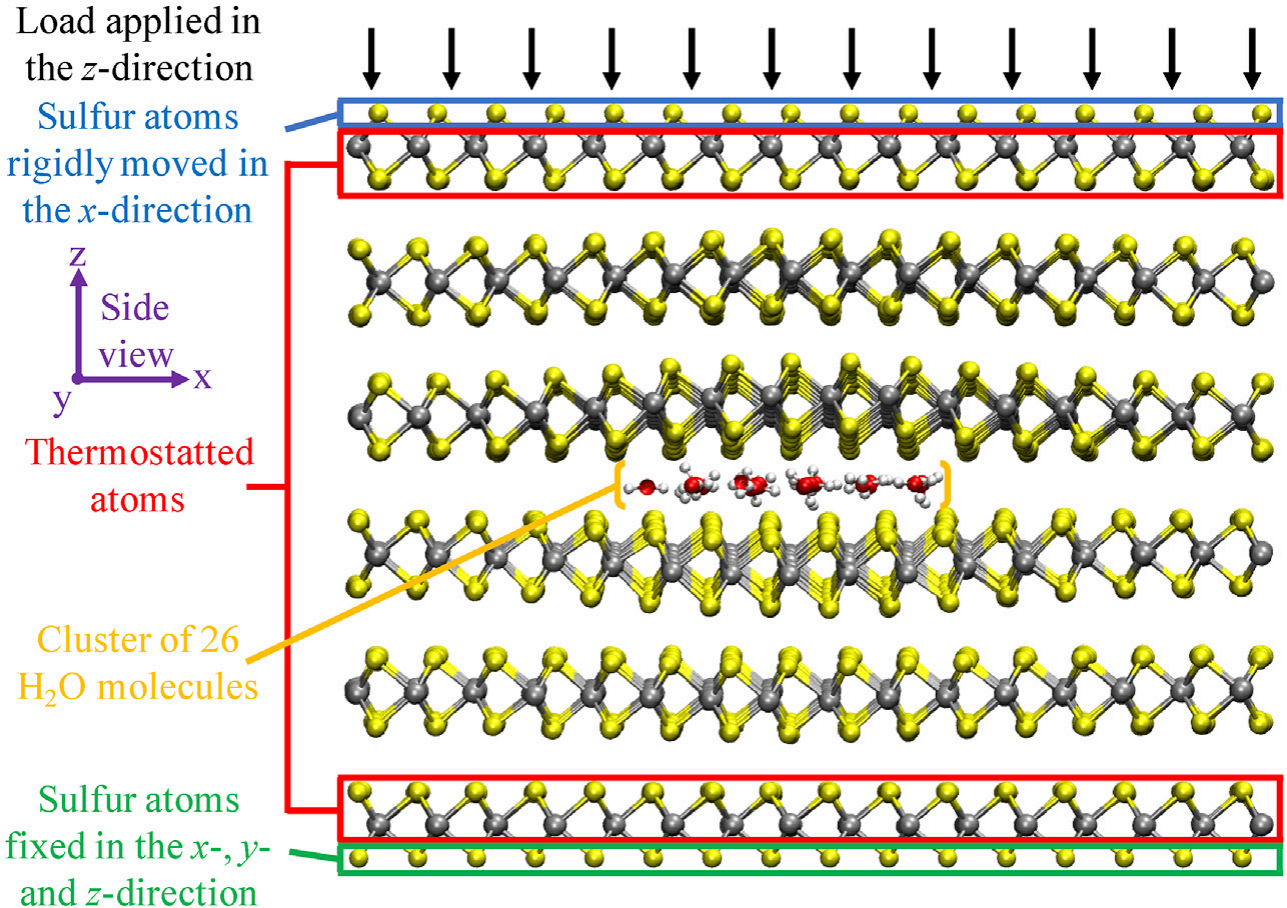
Schematic overview of the computational setup. The side view of the six-layered MoS_2_ structure, with a cluster of 26 H_2_O molecules intercalated in the center interface, is displayed. Molybdenum, sulfur, oxygen, and hydrogen atoms are displayed in silver, yellow, red, and white, respectively. This specific case resembles the system after equilibration at 0 GPa load and 300 K. From this picture, there is a clear structural deformation visible in the MoS_2_ layers directly encapsulating the H_2_O cluster. Figure was obtained with VMD (Humphrey et al., 1996).

Three independent production runs for every configuration were created. In all runs, the bottommost layer of sulfur atoms was held fixed in the *x*-, *y*-, and *z*-directions. Next, the topmost sulfur layer was moved rigidly in the *x*-direction, along the Mo–S bonds, with three different velocities (20.0, 2.0, and 0.2 m/s) and for a total of ∼27.5 Å . For the analysis, the first part of the MD trajectories was discarded and considered as the run-in phase, and only the last ∼22 Å was used, which is the distance based on four periods of the cell in that direction (∼5.5 Å) . The velocities were maintained constant along the trajectories, which was achieved by setting an initial velocity and canceling the force in this direction. On the one hand, this setup allows for the study of the effect of velocity in the sliding direction, and on the other hand, the system keeps the freedom to slide the MoS_2_ layers in the *y*-direction and to breathe in the *z*-direction. The reason to slide along this direction is because this direction represents the highest friction (Claerbout et al., 2019), and therefore, the most interesting dynamics was expected to be observed. Along all runs, a time integration step of 0.1 fs was used. In Figure 2, the computational setup is depicted.

## 3 Results and Discussion

### 3.1 Sliding Dynamics

**FIGURE 3:**
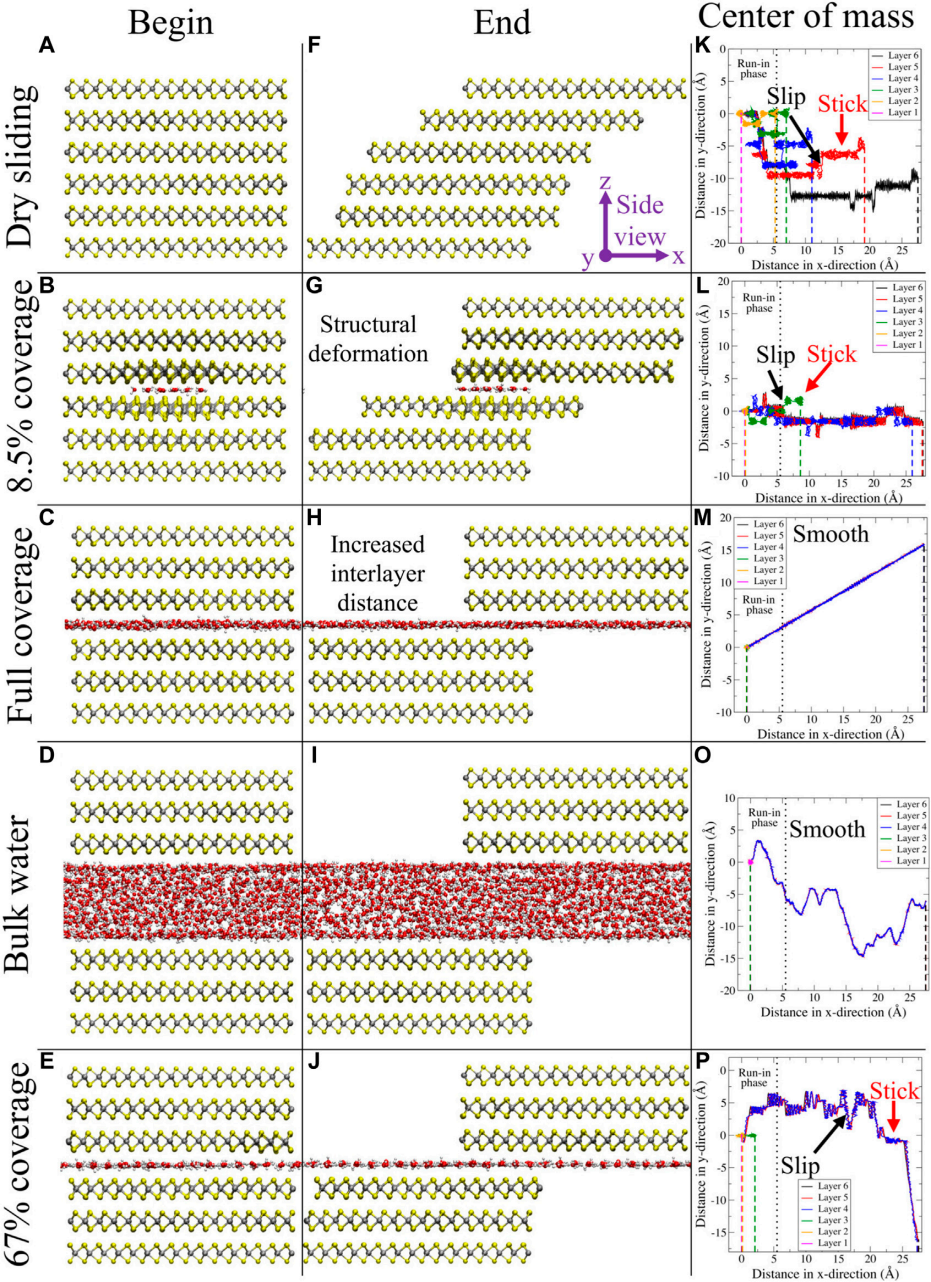
In the first two columns, snapshots for different configurations taken at the beginning **(A–E)** and the end **(F–J)** of the simulations are presented from a side view perspective. Respective configurations are dry sliding, 8.5% coverage, full coverage, bulk water, and 67% coverage. In the third column **(K–O)**, the center of mass for every layer is depicted in the *xy*-plane. The vertical black dotted line in this column represents the end of the run-in phase (which is discarded in the quantitative analysis), while the vertical dashed lines in this column represent the final *x*-position for every layer. Figure was obtained with VMD (Humphrey et al., 1996).

We present a full analysis of the results obtained for 2.0 m/s and describe some of the differences and similarities to sliding with 20.0 and 0.2 m/s. First, a qualitative analysis on the water dynamics is presented, followed by MoS_2_. As stated earlier, the water molecules in the clusters were randomly selected from a melted ice cube. This allows to observe the naturally resulting water network after being transferred from a 3D “free” space to an encapsulated 2D confinement. In general, a flat, single layer of water molecules was formed (see Figures 3A–J). For bulk water, the formation of two water layers was observed, facing MoS_2_ on either side, between which water molecules in a more disordered state were found.

**FIGURE 4:**
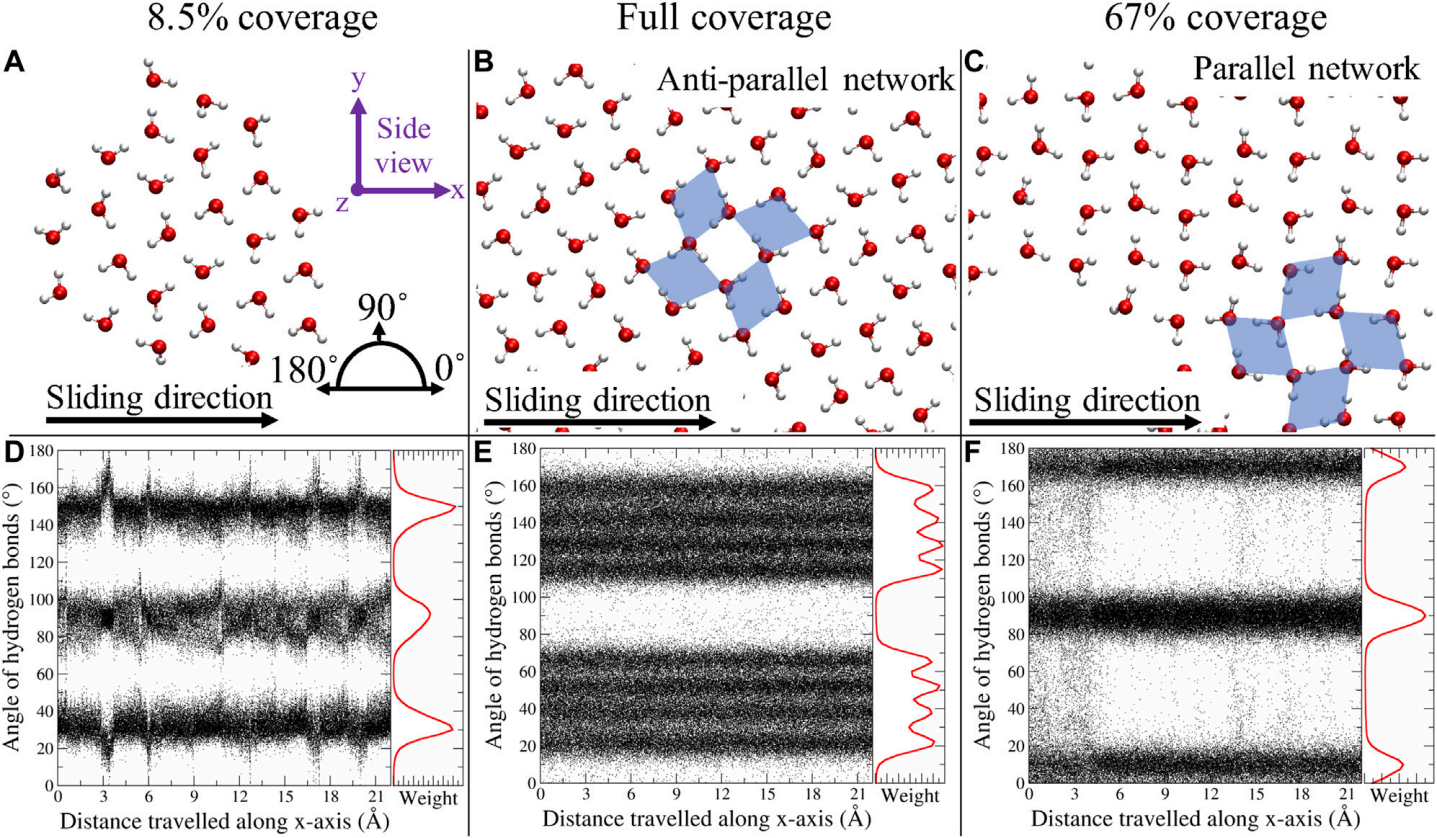
Top view representation of the water network for 8.5%, full, and 67% coverage **(A–C)**. The black arrow represents the sliding direction. The blue diamonds serve as a guide to the eyes in distinguishing the pcd configuration. The bottom row **(D–F)** represents the angles of all H bonds, at every time step, with respect to the *x*-axis; in red, the corresponding histograms are depicted. Figure was obtained with VMD (Humphrey et al., 1996).

In Figures 4A–C, a top view of the water network for three systems is displayed. For up to 8.5% coverage, we found single clusters, kept together by a network of H bonds. In the present study, a hydrogen bond (H bond) is defined as two oxygen atoms, between which there is a maximum distance of 3.5 Å, and where the O–H–O angle is 150°<α<210° (Kwac et al., 2017). Through this H bonds, rings of either three or four molecules were formed. Similar observations were made for increasing water coverage; however, the majority of the rings now consist of four molecules. In fact, a similar type of water network, as described in Kwac et al. (2017), can now be distinguished. This ice-like, the so-called perpendicular crossing diamond network (pcd) consists of rings with four molecules, which are either diamond-like, where the four H bonds are “donated” by two of the water molecules, or square-like, where each water molecule contributes with one H bond. As an example, we put the diamond shapes atop the water network in case of full coverage and 67% coverage. From these observations, it can be concluded that water acts as a solidified film when strictly confined, despite of temperatures above the melting point. Changing the load or the sliding velocity did not alter significantly the formation of the structure.

In an “ideal” water network, the pcd configuration should result in four different H bond angles, two per diamond orientation, which sum up to 180° . We found two main orientations of the water network with respect to the simulation box. We define the first orientation, which was the most common in our sliding MD simulations, as the antiparallel one, where none of the H bonds align along the sliding direction. We define the second orientation, which was only present in a few cases, as the parallel one, where half of the H bonds are oriented practically along the sliding direction. More specifically, in the antiparallel case, these angles are ∼37.5° , ∼52.5° , ∼127.5° , and ∼142.5° (obtained by doing a separate simulation using an ideal “defect-free” water structure without MoS_2_, see the Supplementary Material), whereas in the parallel case, these angles are ∼7.5° , ∼82.5° , ∼97.5° , and ∼172.5° . In Figures 4D–F, the angles of the H bonds with respect to the *x*-axis, present at every time step, are displayed, including the corresponding histograms. In the case of 8.5% coverage or less, three main angles (the middle one with a wider distribution) could be distinguished, which are ∼30° , ∼90° , and ∼150° . This is consistent with the fact that in this case, both three- and four-molecule rings are present. Overall, the H bonds were not aligned along the sliding direction. By increasing the number of water molecules, the pcd network started to form, showing up in six different angles for a water coverage up to 67%, which are ∼30.0° , ∼50.0° , ∼70.0° , ∼110.0° , ∼130.0° , and ∼150.0° (data not shown). Here, the summation of one of the three lower angles combined with one of the three higher ones gives 180° , resulting in a diamond shape. Once more, none of the H bonds were aligned along the sliding direction. For full coverage, four sets of angles were found, again whose sum totals 180° (see Figures 4B,E). On the contrary, in the parallel case as depicted in Figures 4C,F as an example, three angles were found which are ∼10° , ∼90° , and ∼170° , where the central one is the superposition of the two distinct peaks found in the ideal case. The parallel orientation is energetically less favorable (by about 0.04 kcal mol^−1^ Å^−2^, with respect to the static calculations presented in Figure 1) than the parallel one, and generally, along a sliding MD trajectory parallel to antiparallel transitions (but not vice versa) were observed. The deviation between the actual and the expected angles is due to the sliding energy provided to the system, which allows several metastable states where the H bond network experiences a breathing motion. Moreover, the discrete character of the relative orientation of the clusters above/beneath MoS_2_ hints at a rotational disorder energy dependence, which was also revealed by the energy difference between the two ideal cases, referred to above. At any rate, to have a consistent quantitative comparison in terms of the frictional properties, only antiparallel configurations were considered. Finally, considering bulk water, we note that this is the only case where the water actually behaves as a liquid.

Next, we analyze the MoS_2_ dynamics, first from a top view and thereafter from a side view perspective. From the top view, we found a typical characteristic observed in nanoscale friction for 2D solids, the so-called stick–slip dynamics (Lee et al., 2010). In the case of stick–slip, two velocity regimes can be distinguished, a high speed one during which a sudden jump in position occurs and a stick phase. Stick–slip happens as a result of the underlying interactions between two surfaces relative in motion, represented by the potential energy surface (PES), and can be explained using the principles of the Prandtl–Tomlinson (PT) or the more generalized Frenkel–Kontorova model (Socoliuc et al., 2004; Vanossi et al., 2013). Consider a ball being dragged over a PES by a moving spring. When encountering an energy barrier, the ball will stay trapped in the minimum and the spring will be stretched, that is, the stick phase. At a certain point, the energy stored in the spring will be sufficient to pass the energy barrier, the ball “slips” to the next local energy minimum, and as a result, the energy stored in the spring is dissipated. The higher the barrier, the higher will be the dissipation. In general, it is believed that in the temperature-extended PT model and in friction force microscopy experiments, stick–slip is a thermally activated process, whereas in MD simulations, this is only believed to be true for very low sliding speeds (Vanossi et al., 2013).

In our case, the moving spring is indirectly represented by the topmost sulfur layer, which was moved with a constant velocity in the *x*-direction and the van der Waals interactions between the layers. From the above, it becomes clear that whenever the PES has no effective corrugation, stick–slip will not occur. It is found that the corrugation of the PES goes hand in hand with the commensurability of the system (Irving et al., 2017; Wang et al., 2017), as in the case considered in this study. Whenever there is a mismatch, be it a rotational, structural, or a combination of both, the PES flattens out due to the cancellation of forces. In this case, one will observe smooth dynamics, characterized by low energy dissipation.

We observed the stick–slip dynamics for the low water coverage regime (up to 17%), as can be observed in the third column of Figures 3K–O, where the *x* and *y* components of the layer’s center of mass position are reported. From the trajectories, it becomes clear that the slip events can take place in both *x*- and *y*-directions for layers 2–5 (albeit slips are observed more frequently in the *x*-direction). For layer 6, the slip can only occur in the *y*-direction since the velocity in the *x*-direction is held constant. In the Supplementary Material, the reader can find a set of graphs where the profiles of both components of the center of mass position as a function of time are reported. In addition, also the time profiles of the lateral force acting on the top layer can be found in the Supplementary Material. From these graphs, a clear sawtooth-like pattern is present for low water coverages, once more confirming the presence of stick–slip dynamics. All these results are in line with the PES of commensurate MoS_2_, displayed in Figure 1. When the load was increased, stick–slip is enhanced and present for higher water coverage. This observation is in line with earlier results. In 2014, Levita et al. (2014) studied the sliding properties of dry MoS_2_ using first-principle calculations. With an increasing load, the corrugation of the PES increased as a result of changing van der Waals, electrostatic, and Pauli interactions. Moreover, purely by considering the functional form of the force field, especially the LJ part describing the interactions between MoS_2_ layers and between MoS_2_ and water, one could also have predicted this outcome. Increasing the load reduces the interatomic distances and therefore results in a regime with more repulsive LJ interactions. When the velocity was increased, we found that the stick–slip dynamics disappear, even for dry sliding. A possible explanation for this observation is that the adhesion between the layers is reduced due to the increased velocity. This lowers the probability for the atoms to relax to their local minimum energy positions (Onodera et al., 2010). In other words, the layers tend to “fly” over each other, without experiencing a strong interlayer interaction. Although not presented in Figure 3, we also found that the water clusters themselves display stick–slip dynamics up to this low coverage regime.

In general, when the water coverage was increased, we observed a transition to smooth dynamics, both for the MoS_2_ layers and the water clusters. However, in the special case of a parallel water network orientation, stick–slip dynamics persisted again, both in the MoS_2_ and the water clusters. This behavior is also suggested by the static PES profiles (Figure 1), where the PES is displayed for the “ideal” parallel and antiparallel water network compared to “pure” MoS_2_. The antiparallel PES is flat due to lattice incommensurability with MoS_2_. The parallel PES however shows that there is in fact a commensurability present along the sliding direction, where twice the length of the H bond gives the lattice period of MoS_2_.

Next, we analyze the MoS_2_ dynamics from the side view perspective. For low water coverage up to 3%, we observed a “card-deck” shearing behavior, where the bottommost layer remains in place, while the topmost layer moves according to the driving velocity. The intermediate layers present increasing displacements, depending on their relative position in the layered system. At the value of 8.5% water coverage, there is a transition toward a new type of dynamics, where we found that MoS_2_ starts to divide into two blocks, one above and one beneath the water molecules. For moderate coverage (17% and above), the layers in the bottom block, where the bottommost sulfur atoms are fixed in the *x*-, *y*-, and *z*-directions, remain together and do not reposition. Additionally, the layers in the top block also adhere to each other; however, this block slides as a whole according to the driving velocity given to the topmost sulfur layer. This is confirmed by Figures 3A–J, where snapshots at the beginning and end of the trajectory are displayed for different amounts of water coverage. In these specific examples, the water network is oriented in an antiparallel fashion for full coverage, whereas for 67%, this is parallel. We found that for the low–water coverage regime, increasing the velocity slightly shifts the transition from the card-deck shearing to higher water coverage; the same accounts for increasing the load. In other words, card-deck shearing is more pronounced for higher loads and velocities.

Using the PES profiles once more, we can also explain the card-deck shearing vs. block sliding. Let us consider the six layers of MoS_2_ in a minimum energy configuration and start sliding solely the top layer. At a certain moment, an energy barrier will be reached according to the PES, resulting from the interaction with the fifth layer. In this case, instead of passing the barrier, it is energetically more favorable to slide both the top and the fifth layers until the fifth layer reaches the energy barrier from the interaction with the fourth layer. This process continues until the bottom layer is reached, which is static, resulting in the card-deck shearing. We saw that upon introducing water at the interface, the card-deck shearing diminishes until it completely disappears. This behavior can be explained by means of the actual values of the LJ parameters. The interlayer interaction between MoS_2_ is stronger than that between a layer of MoS_2_ and water. Thus, when the top layer slides, the two layers beneath will follow since their adhesion is stronger than that with the water layer. For the low–water coverage regime, the third and fourth layers are still able to interact, albeit less than dry sliding, by means of local bending of the layers around the water cluster. Once the threshold is reached, the layers increase the interlayer distance, instead of local bending, at which point the interaction between water and MoS_2_ dominates.

Finally, we comment on the structural behavior of MoS_2_. For the low-coverage regime, we found a structural distortion of MoS_2_, representing a puckered shape (out-of-plane elastic deformation), most strongly pronounced in the layers directly facing the water. The MoS_2_ layer bends outward from the center interface, creating a locally enhanced interlayer distance. A similar observation was already visible in the computational setup (Figure 2). Increasing the number of water molecules increases the intralayer structural distortion up to a threshold, after which MoS_2_ no longer bends around the water cluster but increases more significantly the interlayer distance of the center interface. This reveals the delicate interplay between the energy cost of the structural distortion and maintaining the interlayer van der Waals interactions. In other words, the LJ interactions between the layers are replaced by LJ contributions between water molecules and the MoS_2_ layers.

### 3.2 Frictional Behavior

**FIGURE 5:**
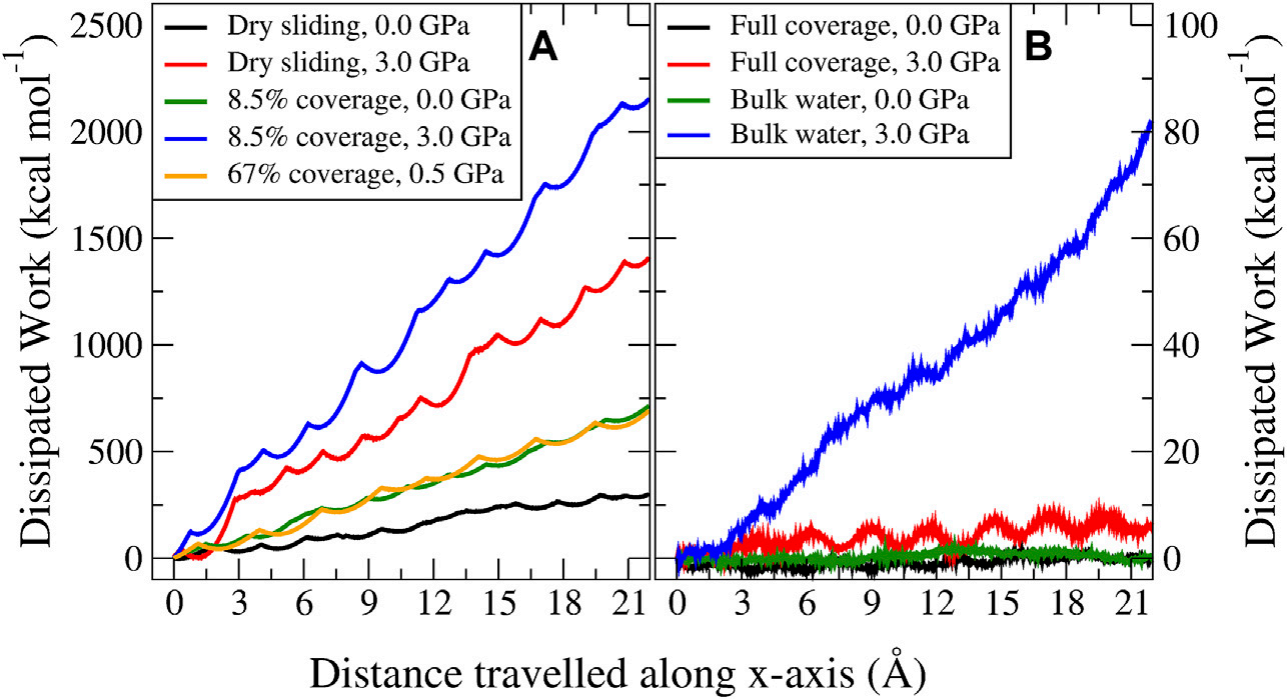
Profiles of the dissipated work for MD trajectories sliding in the *x*-direction. The left panel **(A)** represents the case for dry sliding (black and red) and 8.5% coverage (green and blue) at zero and 3.0 GPa load, respectively, and one case with 67% coverage (orange) at 0.5 GPa load which is characterized by a parallel water network. The right panel **(B)** represents the dissipation at zero and 3.0 GPa load for full coverage with an antiparallel water network (black and red) and bulk water (green and blue).

Using the same analysis as described in Claerbout et al. (2019), we can make an estimation of the dissipated energy as a result of friction coming from the PES corrugation and other channels such as structural distortions. By considering the instantaneous force that is required to slide all the atoms in the uppermost sulfur layer rigidly with a constant velocity along the sliding direction, we can calculate the dissipated work as follows:

$$W=\int_{0}^{\tau }F_{\text{ext}}\left(t\right)vdt,$$ (1)

where $F_{\text{ext}}=-\sum_{i=1}^{N}F_{i,y}$ is the sum of the atomic contributions of the top sulfur layer along the sliding direction. The integral ranges along time τ, the total time of the trajectory, and *v* is the sliding velocity. In Figure 5A, the work profiles at 0 and 3.0 GPa load are displayed for dry sliding and 8.5% water coverage. Furthermore, in this panel, the dissipation is displayed for the parallel water network in the case of 67% coverage. In Figure 5B, the full coverage with an antiparallel orientation and bulk water, again at 0 and 3.0 GPa load, are displayed. From these graphs, several observations can be made. First of all, an increasing load leads to higher dissipation. This observation was expected, and it can be explained with the same argument used earlier. Increasing the load reduces the interlayer distance, resulting in a more repulsive regime of the LJ interactions and a more corrugated PES. A second observation is the dependence of the energy dissipation on the number of water molecules. Starting from dry sliding, increasing the water coverage results in an increasing dissipation. In other words, water acts as a contaminant that increases the frictional forces. This continues up to 3% coverage, after which the dissipation starts to decrease. Reaching moderate coverage (17%), the dissipation falls below dry sliding where the water starts to act as a friction-weakening lubricant. It should be noted that the dissipation behavior follows the same trends as the card-deck shearing vs. block sliding, with respect to the amount of water coverage. These observations can be explained as follows. For dry sliding and low water coverage, a PES corrugation is present, resulting in a significant dissipation during sliding, as explained above. When the number of water molecules is increased in the low coverage regime, an additional dissipation occurs due to the fact that the number of degrees of freedom and the possible channels for energy dissipation increases. This can, for example, be seen by the puckering of the layers in the low water coverage regime. The water causes a puckered shape, resulting in an enhanced strain in the structure, which comes with an energy cost. Next, the puckered shape promotes a deformation of the neighboring layers during sliding. The PES profiles can also explain the frictional behavior in the high coverage regime. There is no effective corrugation in the PES in the case of an antiparallel water network, compared to that of dry MoS_2_; thus, when increasing the water coverage, the dissipated energy decreases. However, in the case of a parallel oriented network, the PES corrugation is of the same order as that of dry MoS_2_, which is translated in a similar dissipation behavior.

Considering different velocities, an interesting behavior was observed. In the case of dry sliding and the low–water coverage regime (below 17%), increasing the velocity decreased the dissipation. Above this coverage threshold, increasing the velocity increased the dissipation, with the most pronounced signature for bulk water. To explain this phenomenon, we divided the coverage into three regimes. The first regime, which we termed the contamination regime, is characterized by low water coverage and is governed by stick–slip dynamics, where, as stated above, a higher velocity results in a possible reduced adhesion and a lower probability for the atoms to move to local minimum energy positions. These factors reduce the energy dissipated during the slip phase. An explanation provided by Zeng et al. (2018) on the frictional velocity dependence of graphene studied by using atomic force microscopy, entailed an aging argument, resulting in a stronger interlayer contact state for lower velocities. Although this case might not directly relate to our computational study (Vanossi et al., 2013), it corroborates the fact that for an enhanced slip, more energy is irreversibly and instantaneously transformed into vibrations and heat. We defined the second regime (ranging roughly between 17% and full coverage) as the solidified water lubrication regime. Here, the lubrication type changes: stick–slip is no longer present and the block sliding mechanism is dominant. Despite the smooth dynamics and the seemingly flat PES compared to dry MoS_2_, some corrugation still remains. Here, thermal activation can be exploited to overcome the low energy barriers, reducing the dissipation. However, with increasing velocity, the system might not explore the local energy minima long enough to benefit from thermal fluctuations toward overcoming the barriers (Szlufarska et al., 2008). Moreover, high velocity regimes are in general further away from equilibrium and characterized by more complex and nonlinear processes, and thereby more dissipative. The third regime (which we termed the liquid water lubrication regime) considers bulk water lubrication, where an enhanced signature of increased dissipation was observed. Apart from the discussion above, here, one should take into account the fact that we are no longer only dealing with solids but also with liquids. Whenever the hydrodynamic fluid film creates enough MoS_2_ interlayer separation, the MoS_2_–water interaction dominates. Liquids are characterized by viscosity, which is known to be strongly affected by the sliding velocity (Vanossi et al., 2013; Ni et al., 2019). In our case, we found out a Newton-like behavior of the fluid characterized by an almost linear increase in the average velocity of the water molecules when moving from the static bottom three layers to the mobile top three layers of MoS_2_ (see the Supplementary Material).

In general, two types of friction can be distinguished, namely, static and kinetic friction. Both types of friction can be calculated *via* the Amontons’ law of friction (Amontons, 1699; Popova and Popov, 2015):

$$F_{∥}=\alpha_{0}+\mu \cdot F_{⊥},$$ (2)

where $F_{∥}$ is the average over the external force in the sliding direction, $\alpha_{0}$ is the frictional force at zero load, *μ* is the coefficient of friction, and $F_{⊥}$ is the normal load applied to the system. Although this law is in general valid only at the macroscopic scale, Mo et al. (2009) have shown that in the case of a single asperity contact, it can work at the nanoscale. In the final part of the analysis, we computed the coefficients of friction present during sliding. Since we calculated the coefficients of friction through a linear fit of the frictional forces experienced by the top layer (which is in constant motion), we are only dealing here with kinetic friction. To obtain the average lateral force, we used a procedure applied before in one of our other studies (Claerbout et al., 2019), the so-called bootstrap method. More details on this averaging technique can be found in the Supplementary Material.

**FIGURE 6:**
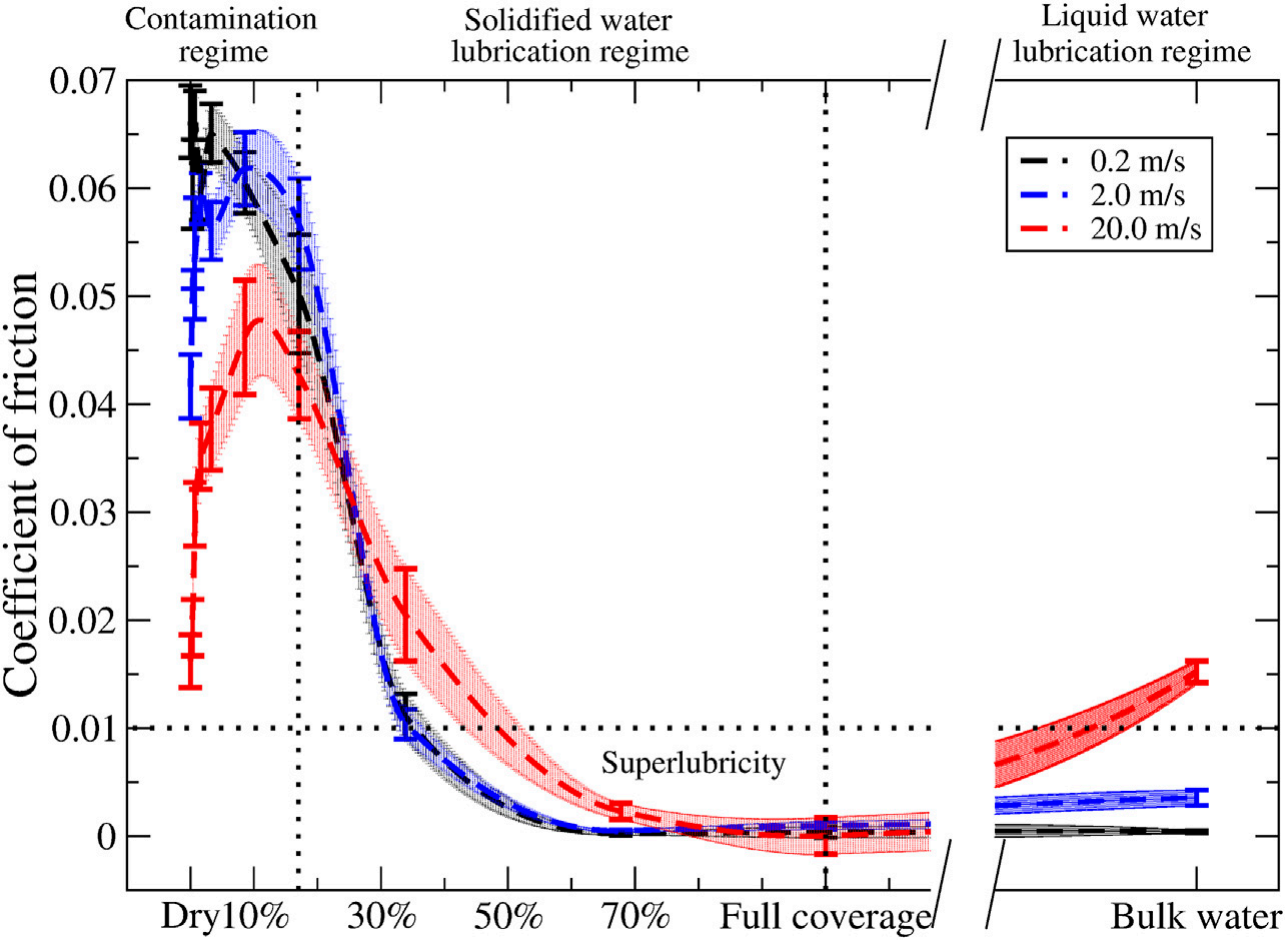
Coefficients of friction for different sliding velocities, 0.2 (black), 2.0 (blue), and 20.0 (red) m/s, plotted vs. water coverage. The dashed lines and the area around it result from akima interpolation and serve as a guide to the eyes. The error bars result from the linear fit taking into account the errors obtained *via* the bootstrap method.

In Figure 6, we present the kinetic coefficients of friction as a function of the water coverage for all three velocities. In this analysis, we selected only the trajectories in which the water network for the high coverage regime was characterized by an antiparallel orientation. This approach is justified since this orientation is more energetically favorable. Again, three frictional regimes can be distinguished. In the first contaminated regime, the coefficient of friction increases with the water coverage, reaching a maximum at a coverage of 3%, after which the coefficient of friction decreases and drops below the dry sliding value for a coverage of 17%. When the water coverage increases further (solidified water lubrication regime), the coefficient of friction monotonically decreases, eventually falling below the superlubricity threshold (COFs below 0.01) (Baykara et al., 2018; Martin and Erdemir, 2018). After 70% water coverage (liquid water lubrication regime), the friction remains seemingly stable till bulk water; at this point, the water molecules are no longer solely in a solid form, but they are also present as a liquid, bearing the usual velocity dependence (Popov, 2017). We found similar trends for the other velocities, albeit that for the contamination regime; the lower velocity has higher coefficients of friction (and vice versa), in agreement with the earlier discussion about energy dissipation.

## 4 Conclusion

In this study, we investigated the frictional properties of bulk commensurate MoS_2_ in the presence of water. By employing nonequilibrium MD simulations, the effects of load, sliding velocity, and the number of water molecules were elucidated. Several conclusions can be drawn from our simulations. First, when placed within a 2D confinement, water acts as a solidified lubricant. The resulting ice-like structure, where the H bonds lead to a characteristic tile-like network, has a discrete (e.g., parallel or antiparallel) orientation above/beneath MoS_2_. Second, two classes of sliding dynamics were distinguished, namely, card-deck shearing vs. block sliding and stick–slip dynamics vs. smooth dynamics. Both show a dependence on the amount of water coverage and the sliding velocity. Within the low water coverage regime, card-deck shearing and stick–slip dynamics are dominant. These types of dynamics are characterized by high dissipative signature and strong load dependence. For moderate to high water coverage, block sliding and smooth dynamics were observed with low energy dissipation and low load dependence. One exception is the metastable case, where the water network aligns along the sliding direction. In this case, a behavior comparable to that of low water coverage is observed. Combining the results above, we found that three lubrication regimes could be identified. In the first one (contamination regime and low water coverage), water acts as a contaminant and friction strengthening was observed with increasing coverage. In the second one (solidified water lubrication regime and high water coverage), water acts as a solid-like lubricant, and superlubricity was observed. In the third one (liquid water lubrication regime and bulk water), water acts as a liquid lubricant, and low coefficients of friction were found, also showing a more pronounced viscosity-like velocity dependence. Finally, we showed and quantified that due to the solidified structure of water, there was a rotational dependence present for the frictional behavior of water between a multilayer system of MoS_2_.

Our results directly contribute to strengthen the general understanding of the nanoscale frictional mechanisms at play when considering MoS_2_ (and 2D materials in general) in the presence of humidity. Once more, it was confirmed that the processes defining friction at this scale are anisotropic and complex in nature. Our results shed light upon the design of nanoscale devices, such as nano- and micro-electromechanical systems (NEMS/MEMS), in which humidity might play an important role, to ramp up the efficiency of the devices. In future work, the scalability of our results to the multi-asperity macroscale will be studied.

## Data Availability

The raw data supporting the conclusion of this article will be made available by the authors, without undue reservation.

## References

[B1] Amontons G. (1699). De la resistance causée dans les machines. Paris: Mémoires de l’Académie royale des sciences, 275–282.

[B2] Arif T. Yadav S. Colas G. Singh C. V. Filleter T. (2019). Understanding the Independent and Interdependent Role of Water and Oxidation on the Tribology of Ultrathin Molybdenum Disulfide (MoS 2 ). Adv. Mater. Inter. 6, 1901246. 10.1002/admi.201901246

[B3] Bampoulis P. Teernstra V. J. Lohse D. Zandvliet H. J. W. Poelsema B. (2016). Hydrophobic Ice Confined between Graphene and MoS2. J. Phys. Chem. C. 120, 27079–27084. 10.1021/acs.jpcc.6b09812

[B4] Baykara M. Z. Vazirisereshk M. R. Martini A. (2018). Emerging Superlubricity: A Review of the State of the Art and Perspectives on Future Research. Appl. Phys. Rev. 5, 041102. 10.1063/1.5051445

[B5] Berman D. Erdemir A. Sumant A. V. (2014). Graphene: a New Emerging Lubricant. Mater. Today 17, 31–42. 10.1016/j.mattod.2013.12.003

[B6] Chhowalla M. Amaratunga G. A. J. (2000). Thin Films of Fullerene-like MoS2 Nanoparticles with Ultra-low Friction and Wear. Nature 407, 164–167. 10.1038/35025020 11001049

[B7] Chhowalla M. Shin H. S. Eda G. Li L.-J. Loh K. P. Zhang H. (2013). The Chemistry of Two-Dimensional Layered Transition Metal Dichalcogenide Nanosheets. Nat. Chem. 5, 263–275. 10.1038/NCHEM.1589 23511414

[B8] Chng E. L. K. Pumera M. (2015). Toxicity of Graphene Related Materials and Transition Metal Dichalcogenides. RSC Adv. 5, 3074–3080. 10.1039/C4RA12624F

[B9] Cho M. H. Ju J. Kim S. J. Jang H. (2006). Tribological Properties of Solid Lubricants (Graphite, Sb2S3, MoS2) for Automotive Brake Friction Materials. Wear. 260, 855–860. 10.1016/j.wear.2005.04.003

[B10] Choi W. Choudhary N. Han G. H. Park J. Akinwande D. Lee Y. H. (2017). Recent Development of Two-Dimensional Transition Metal Dichalcogenides and Their Applications. Mater. Today 20, 116–130. 10.1016/j.mattod.2016.10.002

[B11] Claerbout V. E. P. Polcar T. Nicolini P. (2019). Superlubricity Achieved for Commensurate Sliding: MoS2 Frictional Anisotropy In Silico. Comput. Mater. Sci. 163, 17–23. 10.1016/j.commatsci.2019.03.019

[B13] Curry J. F. Wilson M. A. Luftman H. S. Strandwitz N. C. Argibay N. Chandross M. (2017). Impact of Microstructure on MoS2 Oxidation and Friction. ACS Appl. Mater. Inter. 9, 28019–28026. 10.1021/acsami.7b06917 28758391

[B14] Dudder G. J. Zhao X. Krick B. Sawyer W. G. Perry S. S. (2011). Environmental Effects on the Tribology and Microstructure of MoS2-Sb2O3-C Films. Tribol Lett. 42, 203–213. 10.1007/s11249-011-9764-z

[B15] Evaristo M. Polcar T. Cavaleiro A. (2008). Tribological Behaviour of C-Alloyed Transition Metal Dichalcogenides (TMD) Coatings in Different Environments. Int. J. Mech. Mater. Des. 4, 137–143. 10.1007/s10999-007-9034-2

[B16] Gaur A. P. S. Sahoo S. Ahmadi M. Dash S. P. Guinel M. J.-F. Katiyar R. S. (2014). Surface Energy Engineering for Tunable Wettability through Controlled Synthesis of MoS2. Nano Lett. 14, 4314–4321. 10.1021/nl501106v 25073904

[B17] Hasz K. Ye Z. Martini A. Carpick R. W. (2018). Experiments and Simulations of the Humidity Dependence of Friction between Nanoasperities and Graphite: the Role of Interfacial Contact Quality. Phys. Rev. Mater. 2, 126001. 10.1103/PhysRevMaterials.2.126001

[B18] Holmberg K. Erdemir A. (2017). Influence of Tribology on Global Energy Consumption, Costs and Emissions. Friction 5, 263–284. 10.1007/s40544-017-0183-5

[B19] Humphrey W. Dalke A. Schulten K. (1996). Vmd: Visual Molecular Dynamics. J. Mol. Graphics 14, 33–38. 10.1016/0263-7855(96)00018-5 8744570

[B20] Irving B. J. Nicolini P. Polcar T. (2017). On the Lubricity of Transition Metal Dichalcogenides: an Ab Initio Study. Nanoscale 9, 5597–5607. 10.1039/c7nr00925a 28406512

[B21] Khare H. S. Burris D. L. (2014). Surface and Subsurface Contributions of Oxidation and Moisture to Room Temperature Friction of Molybdenum Disulfide. Tribol Lett. 53, 329–336. 10.1007/s11249-013-0273-0

[B22] Khare H. S. Burris D. L. (2013). The Effects of Environmental Water and Oxygen on the Temperature-dependent Friction of Sputtered Molybdenum Disulfide. Tribol Lett. 52, 485–493. 10.1007/s11249-013-0233-8

[B23] Kwac K. Kim I. Pascal T. A. Goddard W. A. Park H. G. Jung Y. (2017). Multilayer Two-Dimensional Water Structure Confined in MoS2. J. Phys. Chem. C. 121, 16021–16028. 10.1021/acs.jpcc.7b05153

[B24] Late D. J. Liu B. Matte H. S. S. R. Dravid V. P. Rao C. N. R. (2012). Hysteresis in Single-Layer MoS2 Field Effect Transistors. ACS Nano 6, 5635–5641. 10.1021/nn301572c 22577885

[B25] Lee C. Li Q. Kalb W. Liu X. Z. Berger H. Carpick R. W. (2010). Frictional Characteristics of Atomically Thin Sheets. Science 328, 76–80. 10.1126/science.1184167 20360104

[B26] Lee D. Lee H. Lee H. Park J. Y. (2020). Nanotribological Effect of Water Layers Intercalated between Exfoliated MoS2 and Mica. J. Phys. Chem. C. 124, 16902–16907. 10.1021/acs.jpcc.0c01848

[B27] Lee H. Ko J.-H. Choi J. S. Hwang J. H. Kim Y.-H. Salmeron M. (2017). Enhancement of Friction by Water Intercalated between Graphene and Mica. J. Phys. Chem. Lett. 8, 3482–3487. 10.1021/acs.jpclett.7b01377 28697599

[B28] Levita G. Cavaleiro A. Molinari E. Polcar T. Righi M. C. (2014). Sliding Properties of MoS2 Layers: Load and Interlayer Orientation Effects. J. Phys. Chem. C. 118, 13809–13816. 10.1021/jp4098099

[B29] Levita G. Restuccia P. Righi M. C. (2016). Graphene and MoS2 Interacting with Water: A Comparison by Ab Initio Calculations. Carbon 107, 878–884. 10.1016/j.carbon.2016.06.072

[B30] Levita G. Righi M. C. (2017). Effects of Water Intercalation and Tribochemistry on MoS2 Lubricity: An Ab Initio Molecular Dynamics Investigation. ChemPhyschem. 18, 1475–1480. 10.1002/cphc.201601143 28067987

[B31] Li X. Zhu H. (2015). Two-dimensional MoS2: Properties, Preparation, and Applications. J. Materiomics 1, 33–44. 10.1016/j.jmat.2015.03.003

[B32] Luan B. Zhou R. (2016). Wettability and Friction of Water on a MoS2 Nanosheet. Appl. Phys. Lett. 108, 131601. 10.1063/1.4944840

[B33] Luengo G. Israelachvili J. Granick S. (1996). Generalized Effects in Confined Fluids: New Friction Map for Boundary Lubrication. Wear 200, 328–335. 10.1016/S0043-1648(96)07248-1

[B34] Martin J. M. Donnet C. Le Mogne T. Epicier T. (1993). Superlubricity of Molybdenum Disulphide. Phys. Rev. B. 48, 10583–10586. 10.1103/PhysRevB.48.10583 10007345

[B35] Martin J. M. Erdemir A. (2018). Superlubricity: Friction’s Vanishing Act. Phys. Today 71, 40–46. 10.1063/PT.3.3897

[B36] Martyna G. J. Tobias D. J. Klein M. L. (1994). Constant Pressure Molecular Dynamics Algorithms. J. Chem. Phys. 101, 4177–4189. 10.1063/1.467468

[B37] Mo Y. Turner K. T. Szlufarska I. (2009). Friction Laws at the Nanoscale. Nature 457, 1116–1119. 10.1038/nature07748 19242472

[B38] Mutafov P. Evaristo M. Cavaleiro A. Polcar T. (2015). Structure, Mechanical and Tribological Properties of Self-Lubricant W-S-N Coatings. Surf. Coat. Tech. 261, 7–14. 10.1016/j.surfcoat.2014.11.074

[B39] Ni K. Fang H. Yu Z. Fan Z. (2019). The Velocity Dependence of Viscosity of Flowing Water. J. Mol. Liquids 278, 234–238. 10.1016/j.molliq.2019.01.055

[B40] Nian J. Chen L. Guo Z. Liu W. (2017). Computational Investigation of the Lubrication Behaviors of Dioxides and Disulfides of Molybdenum and Tungsten in Vacuum. Friction 5, 23–31. 10.1007/s40544-016-0128-4

[B41] Nicolini P. Polcar T. (2016). A Comparison of Empirical Potentials for Sliding Simulations of MoS2. Comput. Mater. Sci. 115, 158–169. 10.1016/j.commatsci.2016.01.013

[B42] Onodera T. Morita Y. Nagumo R. Miura R. Suzuki A. Tsuboi H. (2010). A Computational Chemistry Study on Friction of H-MoS2. Part II. Friction Anisotropy. J. Phys. Chem. B. 114, 15832–15838. 10.1021/jp1064775 21077588

[B43] Ouyang W. de Wijn A. S. Urbakh M. (2018). Atomic-scale Sliding Friction on a Contaminated Surface. Nanoscale 10, 6375–6381. 10.1039/C7NR09530A 29560981

[B44] Paesani F. Zhang W. Case D. A. Cheatham T. E. III Voth G. A. (2006). An Accurate and Simple Quantum Model for Liquid Water. J. Chem. Phys. 125, 184507. 10.1063/1.2386157 17115765

[B45] Panitz J. K. G. Pope L. E. Lyons J. E. Staley D. J. (1988). The Tribological Properties of MoS2 Coatings in Vacuum, Low Relative Humidity, and High Relative Humidity Environments. J. Vacuum Sci. Tech. A: Vacuum, Surf. Films 6, 1166–1170. 10.1116/1.575669

[B46] Pimentel J. V. Polcar T. Cavaleiro A. (2011). Structural, Mechanical and Tribological Properties of Mo-S-C Solid Lubricant Coating. Surf. Coat. Tech. 205, 3274–3279. 10.1016/j.surfcoat.2010.11.043

[B47] Plimpton S. (1995). Fast Parallel Algorithms for Short-Range Molecular Dynamics. J. Comput. Phys. 117, 1–19. 10.1006/jcph.1995.1039

[B12] Popova E. Popov V. L. (2015). The Research Works of Coulomb and Amontons and Generalized Laws of Friction. Friction 3, 183–190. 10.1007/s40544-015-0074-6

[B48] Popov V. L. (2017). Contact Mechanics and Friction: Physical Principles and Applications. 2 edn. Springer-Verlag Berlin Heidelberg.

[B49] Scharf T. W. Prasad S. V. (2013). Solid Lubricants: a Review. J. Mater. Sci. 48, 511–531. 10.1007/s10853-012-7038-2

[B50] Schönfeld B. Huang J. J. Moss S. C. (1983). Anisotropic Mean-Square Displacements (MSD) in Single-Crystals of 2H- and 3R-MoS2. Acta Crystallogr. Sect B 39, 404–407. 10.1107/S0108768183002645

[B51] Serpini E. Rota A. Ballestrazzi A. Marchetto D. Gualtieri E. Valeri S. (2017). The Role of Humidity and Oxygen on MoS 2 Thin Films Deposited by RF PVD Magnetron Sputtering. Surf. Coat. Tech. 319, 345–352. 10.1016/j.surfcoat.2017.04.006

[B52] Serpini E. Rota A. Valeri S. Ukraintsev E. Rezek B. Polcar T. (2019). Nanoscale Frictional Properties of Ordered and Disordered MoS2. Tribology Int. 136, 67–74. 10.1016/j.triboint.2019.03.004

[B53] Socoliuc A. Bennewitz R. Gnecco E. Meyer E. (2004). Transition from Stick-Slip to Continuous Sliding in Atomic Friction: Entering a New Regime of Ultralow Friction. Phys. Rev. Lett. 92, 134301. 10.1103/PhysRevLett.92.134301 15089616

[B54] Sresht V. Govind Rajan A. Bordes E. Strano M. S. Pádua A. A. H. Blankschtein D. (2017). Quantitative Modeling of MoS2-Solvent Interfaces: Predicting Contact Angles and Exfoliation Performance Using Molecular Dynamics. J. Phys. Chem. C. 121, 9022–9031. 10.1021/acs.jpcc.7b00484

[B55] Szlufarska I. Chandross M. Carpick R. W. (2008). Recent Advances in Single-Asperity Nanotribology. J. Phys. D: Appl. Phys. 41, 123001. 10.1088/0022-3727/41/12/123001

[B56] Tagawa M. Yokota K. Matsumoto K. Suzuki M. Teraoka Y. Kitamura A. (2007). Space Environmental Effects on MoS2 and diamond-like Carbon Lubricating Films: Atomic Oxygen-Induced Erosion and its Effect on Tribological Properties. Surf. Coat. Tech. 202, 1003–1010. 10.1016/j.surfcoat.2007.07.069

[B57] Teo W. Z. Chng E. L. K. Sofer Z. Pumera M. (2014). Cytotoxicity of Exfoliated Transition-Metal Dichalcogenides (MoS2, WS2, and WSe2) Is Lower Than that of Graphene and its Analogues. Chem. Eur. J. 20, 9627–9632. 10.1002/chem.201402680 24976159

[B58] Vanossi A. Manini N. Urbakh M. Zapperi S. Tosatti E. (2013). Colloquium: Modeling Friction: From Nanoscale to Mesoscale. Rev. Mod. Phys. 85, 529–552. 10.1103/RevModPhys.85.529

[B59] Vazirisereshk M. R. Martini A. Strubbe D. A. Baykara M. Z. (2019). Solid Lubrication with MoS2: A Review. Lubricants 7, 57. 10.3390/lubricants7070057

[B60] Wang L. Zhou X. Ma T. Liu D. Gao L. Li X. (2017). Superlubricity of a graphene/MoS2 Heterostructure: a Combined Experimental and DFT Study. Nanoscale 9, 10846–10853. 10.1039/c7nr01451a 28726941

[B61] Watanabe S. Noshiro J. Miyake S. (2004). Tribological Characteristics of WS2/MoS2 Solid Lubricating Multilayer Films. Surf. Coat. Tech. 183, 347–351. 10.1016/j.surfcoat.2003.09.063

[B62] Zeng X. Peng Y. Liu L. Lang H. Cao X. a. (2018). Dependence of the Friction Strengthening of Graphene on Velocity. Nanoscale 10, 1855–1864. 10.1039/c7nr07517k 29309078

[B63] Zhao X. Perry S. S. (2010). The Role of Water in Modifying Friction within MoS2 Sliding Interfaces. ACS Appl. Mater. Inter. 2, 1444–1448. 10.1021/am100090t 20415448

