# Supplementary material for: Exploring Nanoscale Lubrication Mechanisms of Multilayer MoS2 During Sliding: The Effect of Humidity
Source: Front Chem. 2021 Jun 24;9:684441. doi: 10.3389/fchem.2021.684441 (PMC8265273; doi:10.3389/fchem.2021.684441)
Supplement: Supplementary file 1 [file DataSheet1.PDF]

Supporting Information

1 INTRODUCTION

In this Supporting Information we address more details regarding the techniques and methods used. Firstly, we present a benchmark of the force field. Secondly, we describe the bootstrap method. Thirdly, we present the results of the ideal water network used as a reference in the main text. Fourthly, we present the positions of the center of mass of each layer in the x- and y-direction plotted versus time. Fifthly, we present the instantaneous lateral force experienced during the trajectory by the systems in Fig. 3 in the main text. Finally, we analyze the bulk water case in terms of the angular dependence of the water network and we characterize its velocity behavior as a function of the normal coordinate.

2 BENCHMARK OF THE EMPLOYED FORCE FIELD

The force field used to perform the classical molecular dynamics simulations was developed by Srehst *et al.*(Sresht et al., 2017). Here, we will present a benchmark by means of comparison with density functional theory (DFT) (Ref. (Levita et al., 2014)) and experimental data (Ref. (Schönfeld et al., 1983)). Table S1 presents the most relevant structural parameters, comparing the different methods. A more elaborate description of the parameters can be found in Ref. (Nicolini and Polcar, 2016).

	MD	Experimental	DFT
Lateral lattice constant (Å)	3.1741	3.161	3.198
Perpendicular lattice constant (Å)	12.409	12.295	12.454
Mo-S distance (Å)	2.397	2.366	2.425
Mo-S-Mo (ϕ) angle (°)	82.910	83.816	82.504
S-Mo-S (θ) angle (°)	82.910	83.816	82.504
S-Mo-S (ψ) angle (°)	80.284	79.064	80.829
S-Mo-S (ω) angle (°)	135.057	134.634	135.247

Table S1. Structural parameters obtained via the Sresht *et al.* force field (MD) (Sresht et al., 2017) compared to DFT (Levita et al., 2014) and experimental data (Schönfeld et al., 1983).

3 THE BOOTSTRAP METHOD

To obtain the average frictional force from the instantaneous frictional forces experienced while sliding the top layer, we resorted to the random sampling bootstrap method. This method works as follows. First, starting from the original dataset, one generates a new set (with the same number of data points) by randomly selecting points from the original set. During this step, the order of the data may change and some data points may be present multiple times, while others may be missing completely. Next, the average over the new data set is calculated. Finally, the process is repeated 2000 times, after which the final average and the standard deviation are calculated.

4 IDEAL WATER NETWORKS

Here, we present the outcomes for the ideal parallel and antiparallel water networks. We performed two additional MD simulations of 100 ps with the same parameters reported in the main text. Here, no MoS_2 was present (i.e., we simulated the water network only). Moreover, the water layers were fixed in the z-direction to mimic 2D confinement. Although no sliding happened in these simulations, the black arrows

indicate the sliding direction as in Fig. 4 of the main text. Please refer to the main text for the discussion of the outcomes.

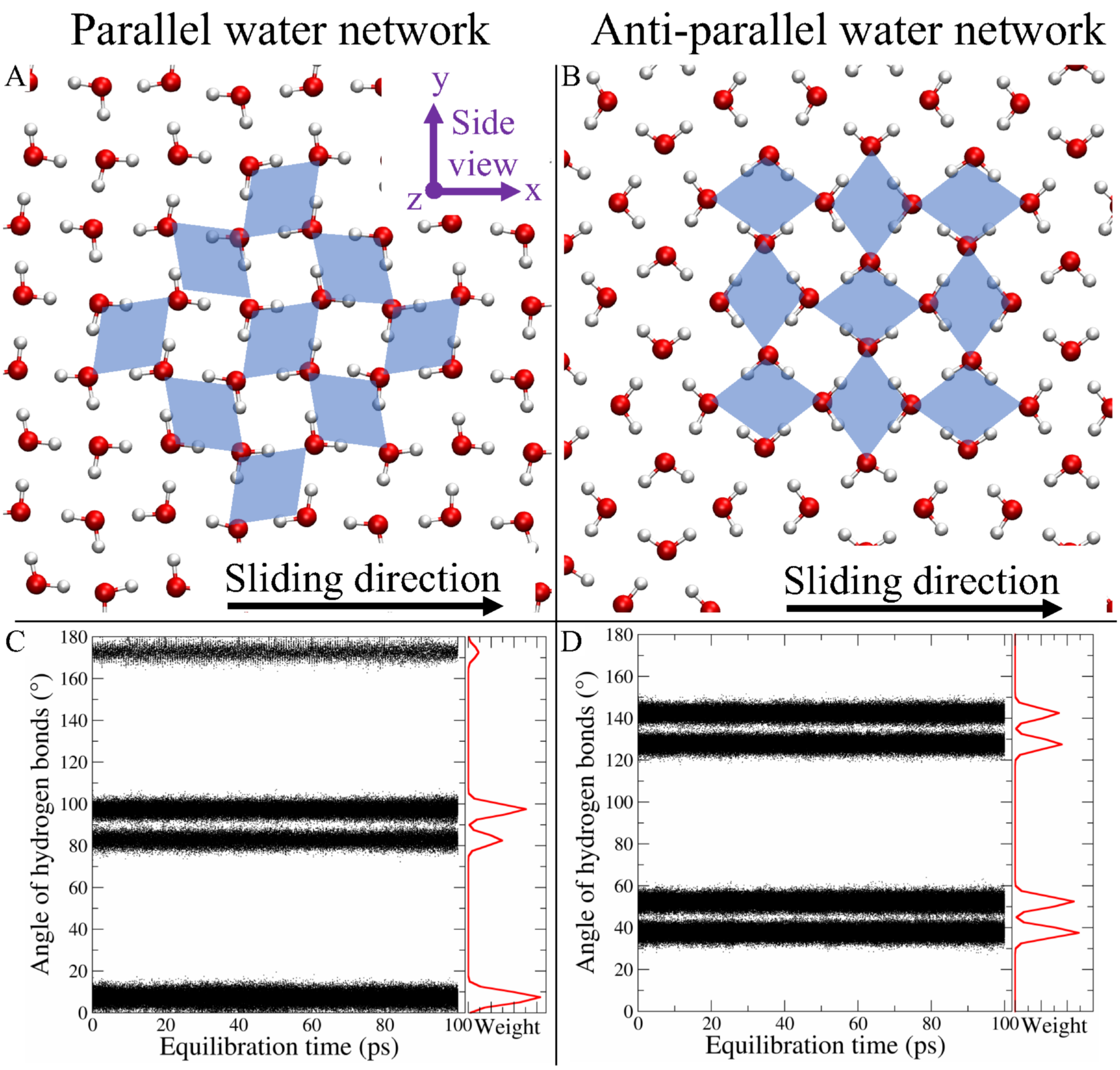

Figure S1. Top view representation of the ideal parallel (panel A) and anti-parallel (panel B) water network. The blue diamond serve as guide for the eye in distinguishing the pcd configuration. The bottom row (panel C-D) represents the angles of all the hydrogen bonds for every time step with respect to the x-axis, in red the corresponding histograms are depicted. Figure obtained with VMD (Humphrey et al., 1996).

5 DYNAMICS OF THE LAYER'S CENTERS OF MASS

Below we present the time profiles of the center of mass of each layer for the trajectories displayed in Fig. 3 in the main text. From these graphs it is clear that, when present, the stick-slip dynamics takes place both in the x- and y-direction.

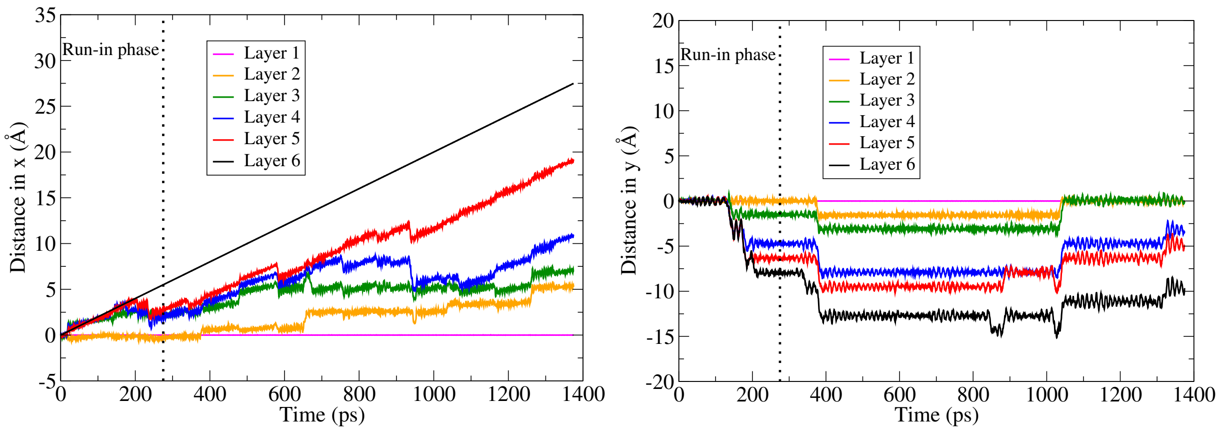

Figure S2. Position of the center of mass in the x- (left panel) and y- (right panel) directions plotted versus time for N = 0 (dry sliding) and 0.0 GPa normal load.

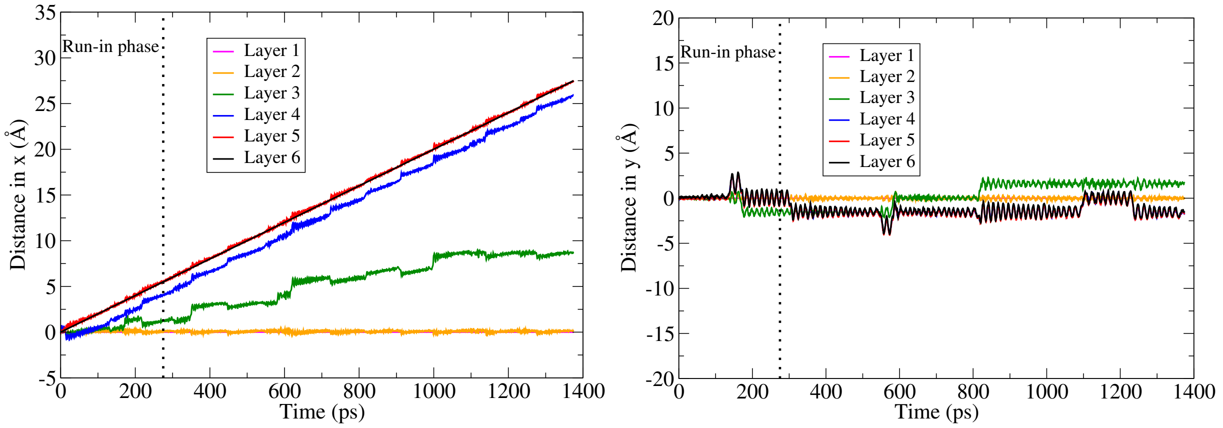

Figure S3. Position of the center of mass in the x- (left panel) and y- (right panel) directions plotted versus time for N = 26 (8.5% coverage) and 0.0 GPa normal load.

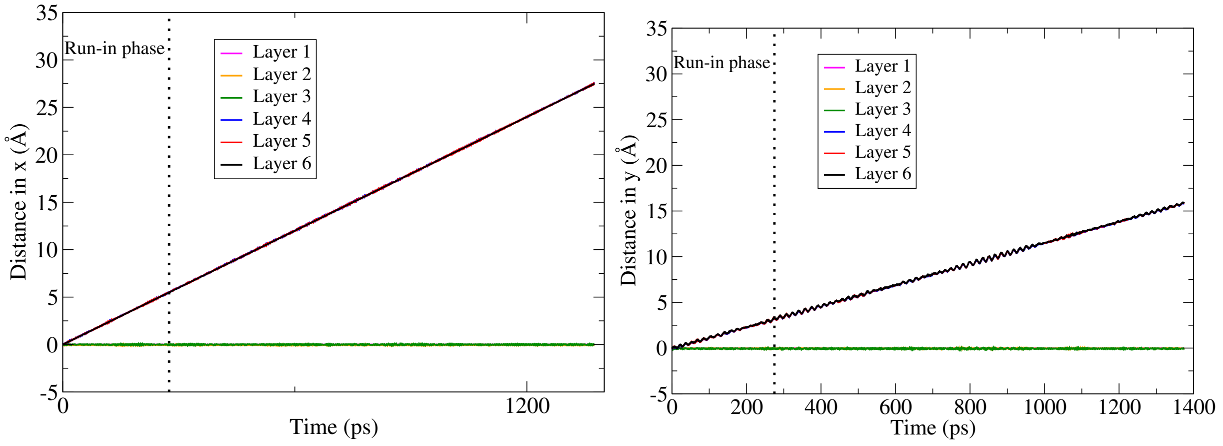

Figure S4. Position of the center of mass in the x- (left panel) and y- (right panel) directions plotted versus time for N = 304 (full coverage) and 0.0 GPa normal load.

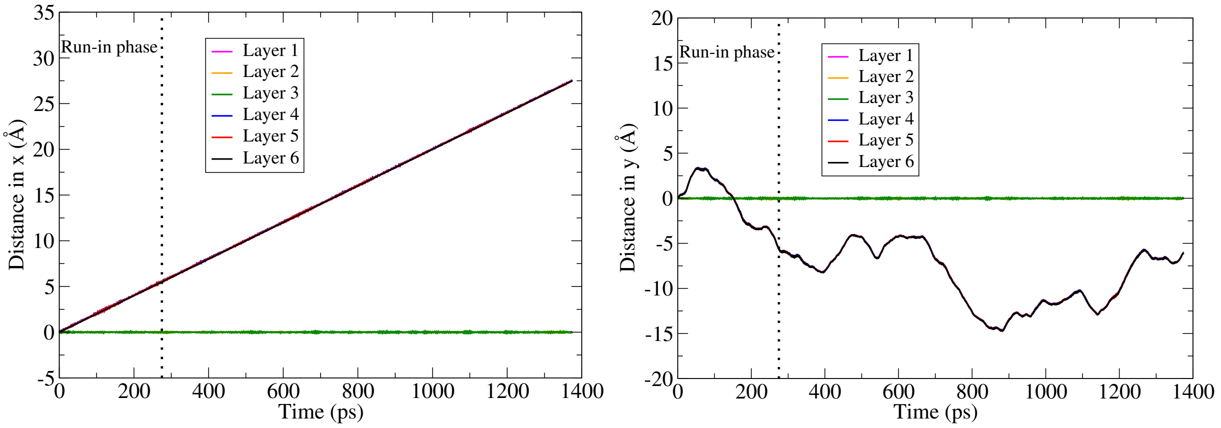

Figure S5. Position of the center of mass in the x- (left panel) and y- (right panel) directions plotted versus time for N = 1236 (bulk water) and 0.0 GPa normal load.

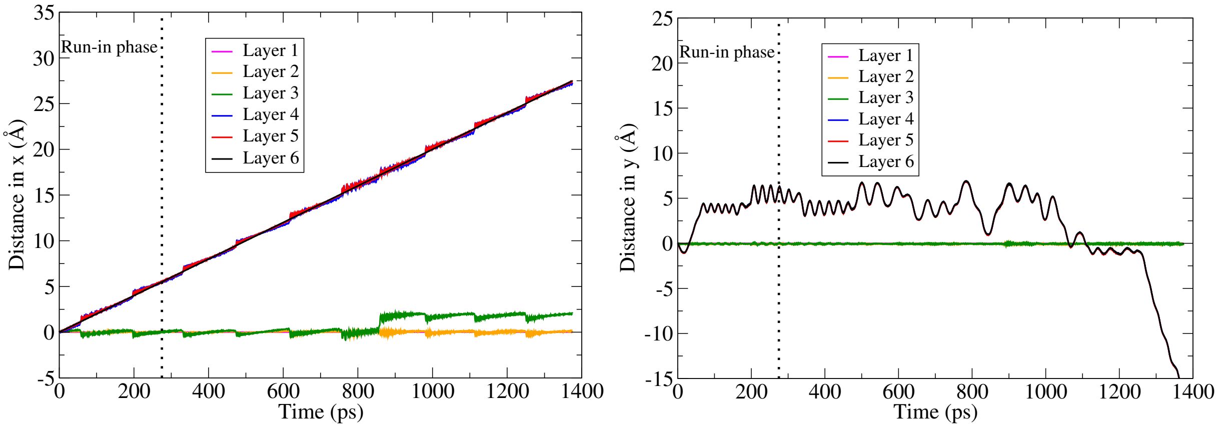

Figure S6. Position of the center of mass in the x- (left panel) and y- (right panel) directions plotted versus time for N = 206 (67 % coverage) and 0.5 GPa normal load.

6 PROFILES OF THE LATERAL FORCE

Here we present the instantaneous *internal* lateral force experienced by the top sulfur layer in the sliding direction (x-direction). In order to keep a constant sliding velocity, an equal and opposite *external* force needs to be provided (which is then used to calculate the friction force). In order to highlight the underlying trends (and therefore average out the large thermal fluctuations), batch averages (with windows of 0.1 Å) are also plotted. A clear saw-tooth shape is present for the cases with N = 0, 26, 206, further confirming the presence of stick-slip dynamics.

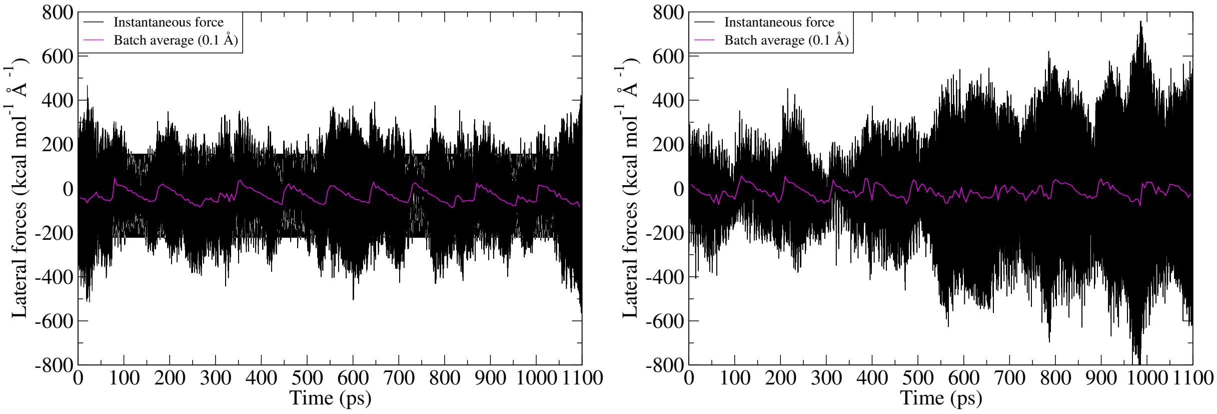

Figure S7. Lateral force experienced during sliding for N = 0 (dry sliding, left panel) and N = 26 (8.5 % coverage, right panel) at 0.0 GPa normal load.

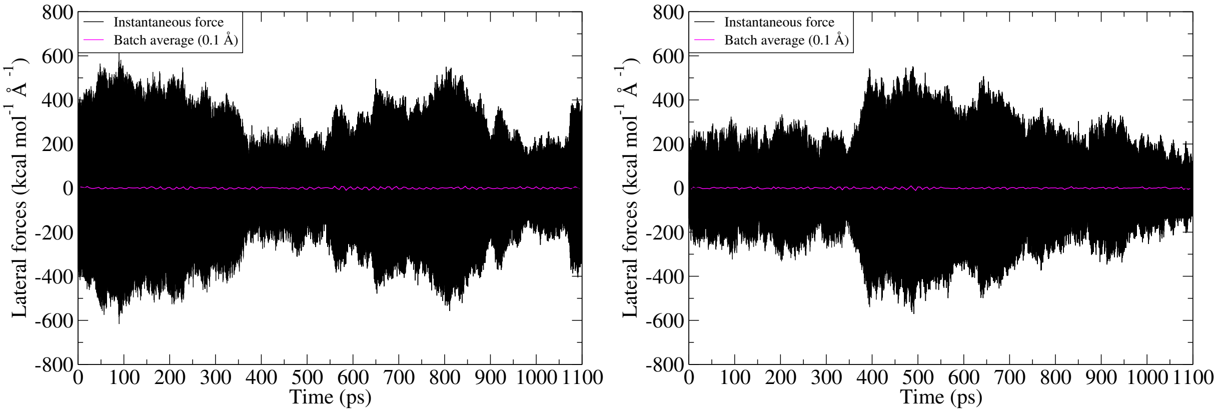

Figure S8. Lateral force experienced during sliding for N = 304 (full coverage, left panel) and N = 1236 (bulk water, right panel) at 0.0 GPa normal load.

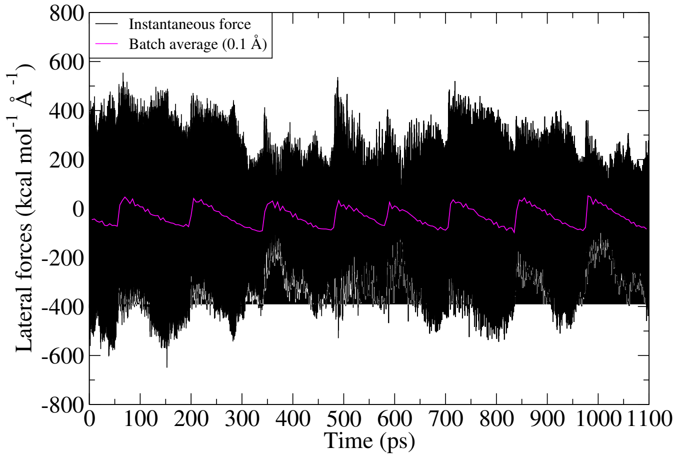

Figure S9. Lateral forces experienced during sliding for N = 206 (67 % coverage) and 0.5 GPa normal load.

7 BULK WATER DYNAMICS

Below we present the analysis of the H-bond angles present in bulk water, similarly to what we report in Fig. 4 in the main text. For reasons of clarity, we report results corresponding to two loads (0.0 and 3.0 GPa). From Fig. S10, we see that, by increasing the normal load, the formation of regularly oriented water layers becomes more pronounced. These water layers are facing the MoS_2 on both sides, which becomes clear from Fig. 3 (panel D and I) in the main text. We also present the distribution of the average velocities of the water molecules as a function of the z-coordinate. Here the thickness of the bulk water was divided into six bins. We present the results for all runs and for two values of normal load (0.0 GPa and 3.0 GPa). From these results it is clear that a linear dependence is present for the velocities based on their position in the z-direction (Newton-like viscosity). The behavior is more pronounced for higher load, where the water molecules are more pronouncedly organized in layers. However, one should keep in mind that large fluctuations are present due to relatively small size of the simulation cell.

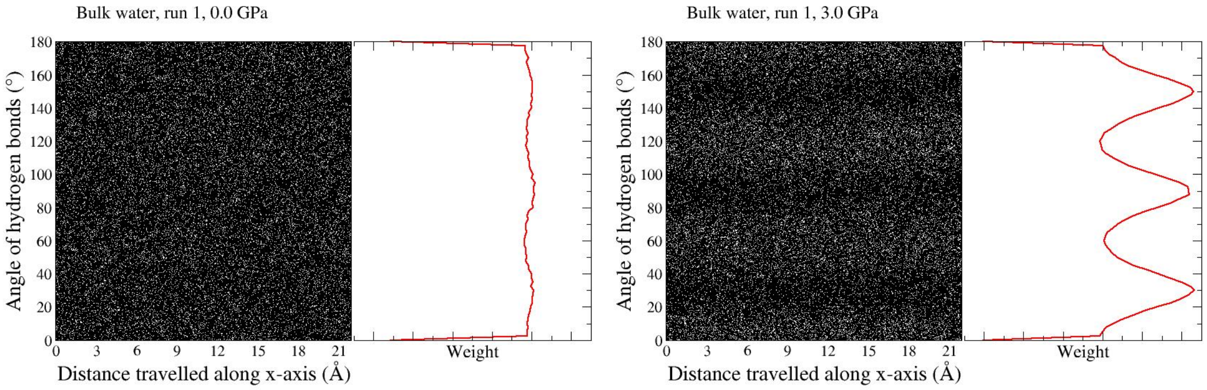

Figure S10. Angle orientation with respect to the x-axis of all hydrogen bonds for bulk water at 0.0 (left panel) and 3.0 GPa (right panel); in red the corresponding histograms are depicted.

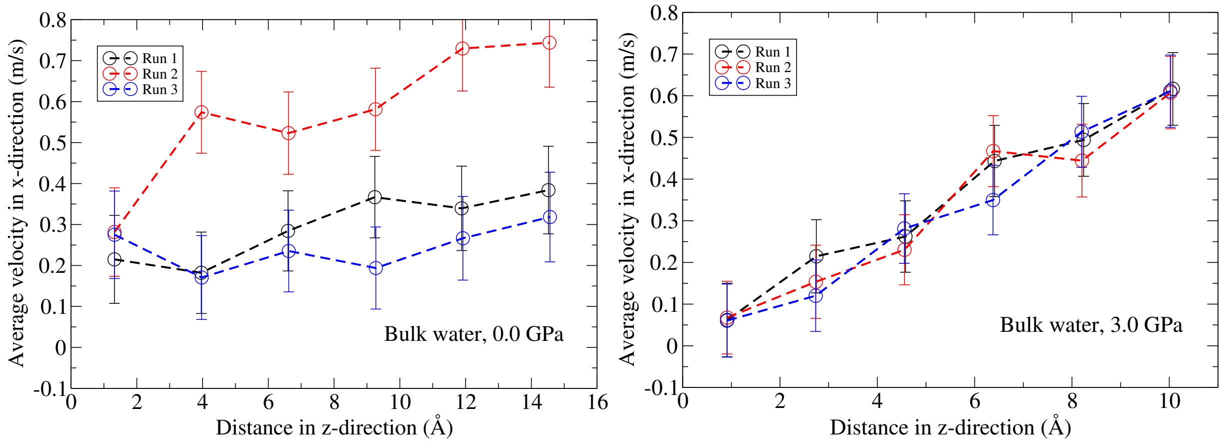

Figure S11. Average velocity of water molecules in the x-direction for bulk water at 0.0 (left panel) and 3.0 GPa (right panel) plotted as a function of the normal coordinate (z-direction). The error bars represent the standard error of the mean.

REFERENCES

Humphrey, W., Dalke, A., and Schulten, K. (1996). Vmd: Visual molecular dynamics. *Journal of Molecular Graphics* 14, 33–38. doi:https://doi.org/10.1016/0263-7855(96)00018-5

Levita, G., Cavaleiro, A., Molinari, E., Polcar, T., and Righi, M. (2014). Sliding properties of MoS_2 layers: Load and interlayer orientation effects. *The Journal of Physical Chemistry C* 118, 13809–13816. doi:10.1021/jp4098099

Nicolini, P. and Polcar, T. (2016). A comparison of empirical potentials for sliding simulations of MoS_2. *Computational Materials Science* 115, 158–169. doi:10.1016/j.commatsci.2016.01.013

Schönfeld, B., Huang, J. J., and Moss, S. C. (1983). Anisotropic mean-square displacements (msd) in single-crystals of 2H- and 3R-MoS_2. *Acta Crystallographica Section B* B39, 404–407. doi:10.1107/S0108768183002645

Sresht, V., Rajan, A. G., Bordes, E., Strano, M. S., Pádua, A. A., and Blankschtein, D. (2017). Quantitative modeling of MoS_2–solvent interfaces: Predicting contact angles and exfoliation performance using molecular dynamics. *The Journal of Physical Chemistry C* 121, 9022–9031. doi:10.1021/acs.jpcc.7b00484
